# Analysis of brain death declaration process and its impact on organ donation in a reference trauma center

**DOI:** 10.31744/einstein_journal/2020AO5448

**Published:** 2020-09-16

**Authors:** Jorge Tadeu Campos Paixão, Vitor Hugo Nunes do Nascimento, Marcela Coutinho Alves, Maria de Fátima Albuquerque Rodrigues, Emanuel de Jesus Soares de Sousa, Bruno Lopes dos Santos-Lobato

**Affiliations:** 1 Universidade do Estado do Pará Belém PA Brazil Universidade do Estado do Pará, Belém, PA, Brazil.; 2 Hospital Metropolitano de Urgência e Emergência Ananindeua PA Brazil Hospital Metropolitano de Urgência e Emergência, Ananindeua, PA, Brazil.

**Keywords:** Brain death, Coma, Tissue and organ procurement, Brain injuries, traumatic, Wound and injuries

## Abstract

**Objective:**

To characterize the processes of brain death diagnosis and organ donation in a reference trauma center.

**Methods:**

Observational and cross-sectional study with patients notified with brain death at a reference trauma center. Data were obtained through the collection of medical records and brain death declaration forms.

**Results:**

One hundred fity-nine patients were notified with brain death, mostly male (82.6%), young adults (97.61%) and victims of brain traumatic injury (93.7%). Median of the total time interval for the diagnosis of brain death was 20.75 hours, with no difference between organ donors and non-donors. We had excessive time intervals on brain death declaration, but without statistical effect on organ donation numbers.

**Conclusion:**

We had low efficacy in brain death declaration based on longer time intervals, with no impact on organ donation.

## INTRODUCTION

Brain death (BD) can be defined as a clinical manifestation of a cerebral catastrophe, characterized by a complete and irreparable neurological damage, recognized by irreversible coma, absence of brain stem reflexes and apnea.^(1)^

The diagnosis of this condition has important medical, ethical and legal implications, since it may influence the withdrawal of all life-sustaining measures or the recovery of organs for transplantation. Brain death declaration is based on clinical examination, according to international standards. There are significant variations in BD declaration methods around the world, and even between different regions and hospitals within the same country.^(2,3)^

All countries demand tests that confirm the absence of brainstem response. However, there are differences regarding the physical examination: in Brazil, two physical examinations are required for BD diagnosis, while Belgium, Netherlands, Norway, Switzerland and most states in the United States proceed with a single physical exam.^(4)^

Apnea test is an essential ancillary test in Brazilian BD declaration protocol, and it is performed worldwide, with differences on pressure of carbon dioxide (PaCO_2_) levels necessary to determine BD: in Brazil, its level is approximately 55mmHg, while in the United States it is about 60mmHg. Also, whereas in Brazil and most of European countries, ancillary exams are needed, they are not usually required in protocols in the United States.^(4)^

In Brazil, from January to December 2017, 10,629 potential donors were notified (patients diagnosed with BD and with no contraindications to organ donation). However, only 3,415 subjects (32%) became effective donors.^(5)^ Major obstacles to higher organ donation numbers in Brazil can be attributed to failure on identification of potential donors, family refusal and clinical contraindications.^(6)^ In 2017, there was an update on Brazilian law regarding BD declaration (Decree 9,175, of October 18, 2017, and Federal Medical Council Resolution 2,173/17, of November 23, 2017) aiming to increase the number of successful organ donations^(7,8)^ – Westphal et al., offer a comprehensive review about these changes.^(9)^

A successful organ donation is associated with short times between first and second clinical examination for BD.^(10)^ Reduction of BD intervals is probably a good strategy to increase the number of organs and tissues available for transplantation.

## OBJECTIVE

To characterize the processes of brain death diagnosis and organ donation in a reference trauma center; to verify the profile of patients who had the diagnosis of brain death; to evaluate the impact of time intervals of brain death declaration on organ donation; and to check predictors of a successful organ donation.

## METHODS

### Study design and subjects

A cross-sectional study was conducted at *Hospital Metropolitano de Urgência e Emergência*, a reference trauma center in Belém (PA), Brazil. Data was obtained by compiling medical records and BD declaration forms in documents of the Organ Procurement Organization (OPO) of patients diagnosed with BD between June 2014 and June 2017. We included all patients aged over 2 years, and excluded patients with missing data on essential hospitalization information. The study was approved by the Research Ethics Committee of the *Universidade do Estado do Pará* (CAAE: 65663717.0.0000.5174, protocol 1.992.783), and since all patients were dead, a waiver of Informed Consent was granted.

Considering data collection took place before the new Brazilian law regarding BD declaration, all cases followed the previous legislation (law 9,434, of February 4, 1997, and Federal Medical Council Resolution 1,480/97).^(11,12)^

### Evaluations

A specific research protocol was used to register data about clinical and epidemiological variables (age, sex, Glasgow Coma Score upon admission, cause of death) and information regarding the steps to BD declaration. Imminent BD upon hospital admission was defined according to de Groot et al.^(13)^ We used the following time variables as indirect measures of the effectiveness of BD declaration: time between hospital admission and intensive care unit (ICU) admission, time between ICU admission and first BD clinical examination, time between first and second BD clinical examination, and time between second BD clinical examination and ancillary test.

We divided organ donation outcomes as strict (“successful” or “unsuccessful” donations) and broad (“successful”, “unsuccessful by family refusal”, “unsuccessful by cardiac arrest” and “unsuccessful by other causes” donations). “Other causes” comprised patients excluded by medical norms (not suitable for organ donation) and other reasons. We defined a “successful organ donation” when an organ was collected of a BD patient to transplantation.

### Statistical analysis

Microsoft Excel 2010 software was used to compile datasheets. The Shapiro-Wilk test was used to evaluate normality of samples. To evaluate categorical variables, the χ^2^ test and Fisher’s exact test were used. For continuous variables, Mann-Whitney test for two independent samples and Kruskal-Wallis test for three or more independent samples were used. Univariate logistic regression analyses were performed for potential predictors of a successful organ donation, with strict organ donation outcome (“successful” or “unsuccessful”) as dependent variable. All statistical inferences were calculated in (SPSS) for Windows, version 23.0 (SPSS Inc., Chicago, USA), adopting a significant p-value ≤0.05.

## RESULTS

### Clinical and epidemiological characteristics

A total of 159 patients were notified with BD in a 3-year period (Table 1). Most BD patients were male (82.6%), young adults (aged 18 to 40 years; n=97; 61%), and victims of traumatic brain injury (93.7%), as expected in a reference trauma center. Children and adolescents accounted for 17.6% (n=28). Approximately one third of patients had imminent BD at hospital admission (34.8%), with no impact on organ donation (χ^2^=0.22; p=0.71). The second BD clinical examination was often performed by neurologists/neurosurgeons (72.3%), and the number of successful organ donations was higher (41.1%) when first BD clinical examination was performed by neurologists/neurosurgeons (χ^2^=4.18; p=0.04). All cases determined as BD in the first clinical examination were confirmed by the second clinical examination. Transcranial Doppler was the ancillary test chosen for all patients.

**Table 1:** Clinical characteristics of brain death patients by strict organ donation outcomes

General characteristics	Overall (n=159)	Successful donation (n=56)	Unsuccessful donation (n=103)	p value
Male sex	137 (86.2)	49 (87.5)	88 (85.4)	0.459^*^
Age, years^†^	28 (20-53)	27 (21-42)	28 (20-40)	0.054^‡^
Imminent BD upon hospital admission	49 (34.8)	19 (37.3)	30 (33.3)	0.386^*^
Cause of death				0.055^*^
Traumatic brain injury	149 (93.7)	56 (100.0)	93 (90.3)	
Stroke	9 (5.7)	0	9 (8.7)	
Metabolic causes	1 (0.6)	0	1 (1.0)	
Neurologist/neurosurgeon as examiner				
At first BD clinical examination	48 (31.0)	23 (41.1)	25 (25.3)	0.041^*^
At second BD clinical examination	107 (72.3)	35 (64.8)	72 (76.6)	0.123^*^
Time between hospital admission and ICU admission, hours^†^	19.8 (11-47)	20.5 (11-48)	19 (10-48)	0.855^‡^
Time between ICU admission and first BD clinical examination, hours^†^	86.6 (43-159)	85 (47-190)	88.2 (41-132)	0.967^‡^
Time between first and second BD clinical examination, hours^†^	10.5 (7-24)	12.4 (7-24)	9 (7-16)	0.878^‡^
Time between second BD clinical examination and ancillary test, hours^†^	10.7 (3-17)	9.8 (2-19)	10.7 (4-15)	0.176^‡^

Resutls expresses as n (%) or median (interquartile range).

* χ^2^ test comparing frequencies of successful and unsuccessful organ donations; ^†^ values in median (interquartile range); ^‡^ Mann-Whitney test comparing medians of successful and unsuccessful organ donations.

BD: brain death; ICU: intensive care unit.

### Impact of time intervals for brain death declaration on organ donation

**Figure 1:**
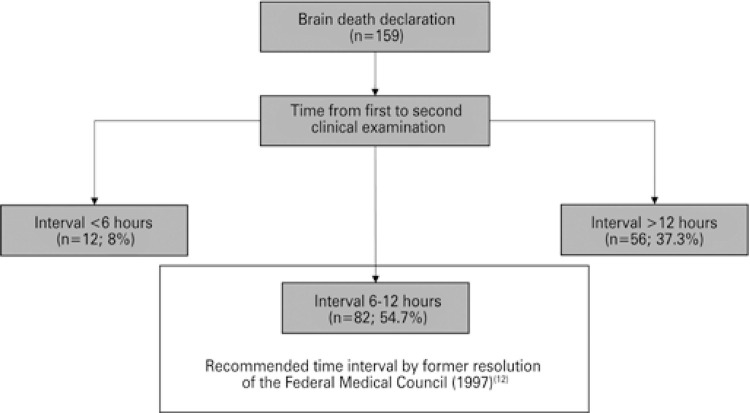
Flow chart of distribution of brain death patients according to time from first to second clinical examinations

Median total BD declaration interval (from first BD clinical examination to final ancillary test) was 20.75 hours (interquartile range 14-29), with no difference between BD patients with successful and unsuccessful organ donations (Mann-Whitney test=2,075, p=0.479). Also, there were no differences between the four analyzed time intervals (time between hospital admission and ICU admission, time between ICU admission and first BD clinical examination, time between first and second BD clinical examination, and time between second BD clinical examination and ancillary test) among BD patients with successful and unsuccessful organ donations. We had long time intervals before first BD clinical examination, mainly the time between ICU admission and first BD clinical examination (median 86.6 hours). Only half of patients had a time between first and second BD clinical examination of approximately 6 to 12 hours, as recommended by the former resolution from Brazilian Federal Medical Council Medicine^(11,12)^ (Figure 1).

We analyzed if different time intervals could increase or reduce the number of successful organ donations. For this, we used two variables: time between first and second BD clinical examination, and time between second BD clinical examination and ancillary test. Both time interval variables were grouped into four quartiles, and the proportion of broad organ donation outcomes were distributed on time intervals groups (Table 2). Regarding time between first and second BD clinical examination, there was no difference on successful organ donations between the shortest (<7 hours) and the longest interval (>15.5 hours; χ^2^=9.86; p=0.36), and proportion of unsuccessful organ donations by family refusal was also similar between extreme time intervals. As to time between second BD clinical examination and ancillary test, the proportion of successful organ donations decreased between the shortest (<2.5 hours) and the longest interval (>15 hours), and the proportion of unsuccessful organ donations by family refusal increased inversely; however, these differences were not statistically significant (χ^2^=6.25, p=0.71).

**Table 2:** Effect of time intervals for brain death declaration on broad organ donation outcomes

	Time between first and second BD clinical examination				
Organ donation outcomes	<7 hours	7-9.5 hours	9.5-15.5 hours	> 15.5 hours	Total
Successful	16 (44.4)	9 (23.1)	13 (34.2)	15 (40.5)	53 (35.3)
Unsuccessful – family refusal	17 (47.2)	25 (64.1)	18 (47.4)	15 (40.5)	75 (50.0)
Unsuccessful – cardiac arrest	0	2 (5.1)	3 (7.9)	1 (2.7)	6 (4.0)
Unsuccessful – other causes	3 (8.3)	3 (7.7)	4 (10.5)	6 (16.2)	16 (10.7)
Total	36 (24.0)	39 (26.0)	38 (25.3)	37 (24.7)	150 (100.0)
	**Time between second BD clinical examination and ancilllary test**				
Organ donation outcomes	<2.5 hours	2.5-7 hours	7-15 hours	>15 hours	Total
Successful	13 (40.6)	18 (46.2)	12 (36.4)	11 (30.6)	54 (38.6)
Unsuccessful – family refusal	15 (46.9)	16 (41.0)	17 (51.5)	21 (58.3)	69 (49.3)
Unsuccessful – cardiac arrest	3 (9.4)	1 (2.6)	1 (3.0)	1 (2.8)	6 (4.3)
Unsuccessful – other causes	1 (3.1)	4 (10.3)	3 (9.1)	3 (8.3)	11 (7.9)
Total	32 (22.9)	39 (27.9)	33 (23.6)	36 (25.7)	140 (100.0)

Results expresses as n (%).

Values represent proportion and number of brain death patients distributed into quartiles of time intervals (time between two clinical examinations, and time between second clinical examination and ancillary test) according to the broad organ donation outcomes.

BD: brain death.

### Predictors of a successful organ donation after brain death

Univariate logistic regression analyses were performed for the role of predictors (age, sex, Glasgow coma score upon admission and time intervals) on strict organ donation outcomes, and only age was inversely associated to odds of a successful organ donation (B coefficient -0.027; odds ratio 0.973; 95% confidence interval 0.94-0.99; p=0.027) (Table 3).

**Table 3:** Univariate analysis of predictors of a successful organ donation after brain death

Predictors	B	OR	95%CI	p value
Sex				
Female	Reference			
Male	0.177	1.19	0.45-3.12	0.719
Age at time of study	-0.027	0.97	0.94-0.99	0.027
Glasgow coma scale 3 upon admission	0.172	1.18	0.58-2.43	0.639
Time between hospital admission and ICU admission	0.176	1.19	0.4-3.48	0.747
Time between ICU admission and first BD clinical examination	0.184	1.2	0.36-3.95	0.763
Time between first and second BD clinical examination	0.051	1.05	0.29-3.7	0.937
Time between second BD clinical examination and ancillary test	-0.234	0.79	0.43-1.44	0.444

B: regression coefficient; BD: brain death; OR: odds ratio; 95%CI: confidence interval 95%.

## DISCUSSION

Our results showed our BD patients were mostly young male adults who suffered traumatic brain injury. Previous studies carried out in Brazil described young male adults as the main group associated to BD, but traumatic brain injury was not the main cause of death of these patients.^(14-17)^ Since our sample was collected at a reference trauma center, our results may have a selection bias.

Approximately one third of these patients had been admitted in imminent BD, a concept created to enhance clinical recognition of potential organ donors, with high sensibility and low specificity to predict BD.^(13,18)^ In our sample, the hospital admission with Glasgow coma scale score 3 did not influence the number of successful organ donations. If specific measures of BD elucidation for family and intensive care were used in immediate BD patients, the number of donors could be higher.

Time intervals for BD declaration in our study were longer than expected, and we attributed this fact to specific problems of the reference center (few neurologists and neurosurgeons, low level of medical training on BD declaration, and low availability of ancillary tests). These data helped the organization improve its BD declaration protocol and to enhance the availability of ancillary tests.

However, there is great variability regarding these time intervals worldwide. Previous works described time between first and second BD clinical examination from 8.9 hours^(17)^ to 19.2 hours,^(10)^ and total BD declaration interval was reported as 14.4 hours.^(17)^ A longer total BD declaration interval is associated with more cardiac arrests,^(19)^ higher costs in intensive care and poor quality of organs designated for transplant,^(20,21)^ and a shorter time interval between admission to BD declaration increases the number of organs transplanted per donor.^(22)^ Also, cold ischemia time longer than 24 hours are associated with lower kidney graft survival.^(23)^

Also, we must consider the great variability in the process of BD declaration worldwide.^(4)^ A recent review reported low homogeneity in the process of BD declaration in 91 countries, with different protocols for apnea testing, requirement and type of ancillary test, number and qualification of physicians, and criteria applied to children.^(24)^

Our study showed time between first and second BD clinical examination had no effect on organ donation outcome, both on comparison of proportion of donors in distinct interval quartiles and on univariate logistic regression. This result differs from conclusions of a previous work analyzing the effect of this time interval on organ donation on 1,311 BD patients.^(10)^ They demonstrated longer intervals between two BD examinations caused reduction in proportion of donors, increase in family refusal of organ donation, and more cardiac arrest. We attributed these divergent results to differences on sample size and variability of BD declaration methodology.

Our study has important limitations. A small sample size and recruitment from one single center that receives mainly trauma patients may impair generalizability of results. Low proportion of donors (35.2%) may also interfere in final results. As strength, our work is the first Brazilian study that explored time intervals for BD declaration and their impact on organ donation.

## CONCLUSION

We observed low efficacy of brain death declaration based on longer time intervals for brain death declaration, with no impact on organ donation. Reduction in time between two clinical examinations for brain death and a fast execution of ancillary tests should be priorities at hospitals to enhance the quality of brain death declaration.

## References

[1] 1. Machado C. Diagnosis of brain death. Neurol Int. 2010;2(1):e2. 10.4081/ni.2010.e2 PMC3093212 21577338

[2] 2. Escudero D, Matesanz R, Soratti CA, Flores JI; nombre de la Red/Consejo Iberoamericano de Donación Y Transplante. [General considerations on brain death and recommendations on the clinical decisions after its diagnosis. Red/Consejo Iberoamericano de Donación y Trasplante]. Med Intensiva. 2009;33(9):450-4. Review. Spanish. 10.1016/j.medin.2009.06.004 19922827

[3] 3. Escudero D, Valentín MO, Escalante JL, Sanmartín A, Perez-Basterrechea M, de Gea J, et al. Intensive care practices in brain death diagnosis and organ donation. Anaesthesia. 2015;70(10):1130-9. 10.1111/anae.13065 26040194

[4] 4. Vinhas AM, Gros AM, Favero B, Bom JM, Faleiro L, Ceccato ME, et al. Evaluation of brain death diagnosis around the world. Acta Med. 2018; 39(1):387-97.

[5] 5. Registro Brasileiro de Transplantes (RBT). Dimensionamento dos Transplantes no Brasil e em cada estado (2010-2017) [Internet]. RBT; 2017 [citado 2018 Jul 13]. Disponível em: http://www.abto.org.br/abtov03/Upload/file/RBT/2017/rbt-imprensa-leitura-compressed.pdf

[6] 6. Dell Agnolo CM, de Freitas RA, Toffolo VJ, de Oliveira ML, de Almeida DF, Carvalho MD, et al. Causes of organ donation failure in Brazil. Transplant Proc. 2012;44(8):2280-2. 10.1016/j.transproceed.2012.07.133 23026573

[7] 7. Brasil. Presidência da República. Casa Civil. Subchefia para Assuntos Jurídicos. Decreto nº 9175, de 18 de outubro de 2017. Regulamenta a Lei nº 9.434, de 4 de fevereiro de 1997, para tratar da disposição de órgãos, tecidos, células e partes do corpo humano para fins de transplante e tratamento [Internet]. Brasília (DF): Casa Civil; 2017 [citado 2019 Jul 10]. Disponível em: http://www.planalto.gov.br/ccivil_03/_Ato2015-2018/2017/Decreto/D9175.htm7

[8] 8. Conselho Federal de Medicina (CFM). Resolução CFM nº 2173, de 23 de novembro de 2017. Define os critérios do diagnóstico de morte encefálica [Internet]. Brasília (DF): CFM; 2017 [citado 2019 Jul 10]. Disponível em: https://sistemas.cfm.org.br/normas/visualizar/resolucoes/BR/2017/2173

[9] 9. Westphal GA, Veiga VC, Franke CA. Diagnosis of brain death in Brazil. Rev Bras Ter Intensiva. 2019;31(3):403-9. 10.5935/0103-507X.20190050 PMC7005965 31618361

[10] 10. Lustbader D, O’Hara D, Wijdicks EF, MacLean L, Tajik W, Ying A, et al. Second brain death examination may negatively affect organ donation. Neurology. 2011;76(2):119-24. 10.1212/WNL.0b013e3182061b0c 21172836

[11] 11. Brasil. Presidência da República. Casa Civil. Subchefia para Assuntos Jurídicos. Lei nº 9.434, de 4 de fevereiro de 1997. Dispõe sobre a remoção de órgãos, tecidos e partes do corpo humano para fins de transplante e tratamento e dá outras providências [Internet]. Brasília (DF): Casa Civil; 1997 [citado 2019 Jul 10]. Disponível em: http://www.planalto.gov.br/ccivil_03/LEIS/L9434.htm

[12] 12. Conselho Federal de Medicina (CFM). Resolução CFM nº 1.480/1997. Define critérios para diagnóstico de morte encefálica [Internet]. Brasília (DF): CFM; 1997 [citado 2019 Jul 10]. Disponível em: http://www.portalmedico.org.br/resolucoes/CFM/1997/1480_1997.htm

[13] 13. de Groot YJ, Jansen NE, Bakker J, Kuiper MA, Aerdts S, Maas AI, et al. Imminent brain death: point of departure for potential heart-beating organ donor recognition. Intensive Care Med. 2010;36(9):1488-94. 10.1007/s00134-010-1848-y PMC2921050 20232039

[14] 14. Rodrigues SL, Ferraz Neto JB, Sardinha LA, Araujo S, Zambelli HJ, Boin IF, et al. Profile of effective donors from organ and tissue procurement services. Rev Bras Ter Intensiva. 2014;26(1):21-7. 10.5935/0103-507X.20140004 PMC4031888 24770685

[15] 15. Pessoa JL, Schirmer J, Roza BA. Evaluation of the causes for family refusal to donate organs and tissue. Acta Paul Enferm. 2013;26(4):323-30.

[16] 16. Souza BS, Lira GG, Mola R. Notification of brain death in the hospital. Rev Rene. 2015;16(2):194-200.

[17] 17. Rocha DF, Nothen RR, Santos SR, Oliveira PC. Evaluation of the time of diagnosis of brain deaths reported to the Transplant Center of Rio Grande do Sul. Sci Med. 2015;25(3):ID21328.

[18] 18. Zappa S, Fagoni N, Bertoni M, Selleri C, Venturini MA, Finazzi P, Metelli M, Rasulo F, Piva S, Latronico N; Imminent Brain Death (IBD) Network Investigators. Determination of Imminent Brain Death Using the Full Outline of Unresponsiveness Score and the Glasgow Coma Scale: A Prospective, Multicenter, Pilot Feasibility Study. J Intensive Care Med. 2020;35(2):203-7. 10.1177/0885066617738714 29084482

[19] 19. Westphal GA, Slaviero TA, Montemezzo A, Lingiardi GT, de Souza FC, Carnin TC, et al. The effect of brain death protocol duration on potential donor losses due to cardiac arrest. Clin Transplant. 2016;30(11):1411-6. 10.1111/ctr.12830 27532678

[20] 20. Marinho A, Cardoso SS, Almeida VV. Os transplantes de órgãos nos Estados Brasileiros [Internet]. Rio de Janeiro (RJ): IPEA; 2007 [citado 2017 Nov 15]. Disponível em: http://repositorio.ipea.gov.br/bitstream/11058/1575/1/TD_1317.pdf

[21] 21. Wong J, Tan HL, Goh JP. Management of the brain dead organ donor. Trends Anaesthesia Critical Care. 2017;13:6-12.

[22] 22. Resnick S, Seamon, MJ, Holena D, Pascual J, Reilly PM, Martin ND. Early declaration of death by neurologic criteria results in greater organ donor potential. J Surg Res. 2017;218:29-34. 10.1016/j.jss.2017.05.032 PMC5803185 28985863

[23] 23. Baptista A, Silva HT Jr, Pestana JO. Influence of deceased donor hemodynamic factors in transplant recipients renal function. J Bras Nefrol. 2013;35(4):289-98. 10.5935/0101-2800.20130048 24402109

[24] 24. Wahlster S, Widjicks EF, Patel PV, Greer DM, Hemphill JC 3rd, Carone M, et al. Brain death declaration: Practices and perceptions worldwide. Neurology. 2015;84(18):1870-9. 10.1212/WNL.0000000000001540 PMC4433464 25854866

