# Supplementary material for: Analysis of brain death declaration process and its impact on organ donation in a reference trauma center
Source: Einstein (Sao Paulo). 2020 Sep 16;18:eAO5448. doi: 10.31744/einstein_journal/2020AO5448 (PMC9586427; doi:10.31744/einstein_journal/2020AO5448)
Supplement: Translation

# Translation: Análise do processo de declaração de morte encefálica e seu impacto na doação de órgãos em um centro de referência em trauma

**Authors:** Jorge Tadeu Campos Paixão, Vitor Hugo Nunes do Nascimento, Marcela Coutinho Alves, Maria de Fátima Albuquerque Rodrigues, Emanuel de Jesus Soares de Sousa, Bruno Lopes dos Santos-Lobato

**Affiliations:** 1 Brasil Universidade do Estado do Pará, Belém, PA, Brasil.; 2 Brasil Hospital Metropolitano de Urgência e Emergência, Ananindeua, PA, Brasil.

**Keywords:** Morte encefálica, Coma, Obtenção de tecidos e órgãos, Lesões encefálicas traumáticas, Ferimentos e lesões

## Abstract

**Objetivo:**

Caracterizar os processos de diagnóstico de morte encefálica e doação de órgãos em um centro de referência de trauma.

**Métodos:**

Estudo observacional e transversal com pacientes notificados com morte encefálica em um centro de referência de trauma. Os dados foram obtidos por meio da coleta de prontuários e formulários de declaração de morte encefálica.

**Resultados:**

Foram notificados com morte encefálica 159 pacientes, com prevalência do sexo masculino (82,6%), adultos jovens (97,61%) e vítimas de traumatismo craniencefálico (93,7%). A mediana do intervalo total de tempo para o diagnóstico de morte encefálica foi de 20,75 horas, sem diferença entre doadores de órgãos e não doadores. Tivemos intervalos de tempo excessivos na declaração de morte encefálica, mas sem efeito estatístico no número de doações de órgãos.

**Conclusão:**

Foi baixa a eficácia na declaração de morte encefálica com base em intervalos de tempo mais longos, sem impacto na doação de órgãos.

## INTRODUÇÃO

A morte encefálica (ME) pode ser definida como a manifestação clínica de uma catástrofe cerebral, caracterizada por dano neurológico completo e irreparável, reconhecido por coma irreversível, ausência de reflexos no tronco encefálico e apneia.^(1)^

O diagnóstico dessa condição tem implicações médicas, éticas e legais importantes, pois pode influenciar na retirada de todas as medidas de sustentação da vida ou na recuperação de órgãos para transplante. A declaração de ME é baseada no exame clínico, de acordo com padrões internacionais. Existem variações significativas nos métodos de declaração da ME em todo o mundo, e ainda entre diferentes regiões e hospitais no mesmo país.^(2,3)^

Todos os países exigem exames que confirmem a ausência de resposta do tronco cerebral. No entanto, existem diferenças quanto ao exame físico: no Brasil, são necessários dois exames físicos para o diagnóstico de ME, ao passo que, na Bélgica, na Holanda, na Noruega, na Suíça e na maioria dos estados dos Estados Unidos, é realizado um único exame físico.^(4)^

O teste de apneia é um exame complementar essencial no protocolo brasileiro de declaração de ME, sendo realizado em todo o mundo, com diferenças nos níveis de pressão de dióxido de carbono (PaCO_2_) necessários para determinar a ME: no Brasil, seu nível é de cerca de 55mmHg, ao passo que, nos Estados Unidos, é de cerca de 60mmHg. Além disso, enquanto no Brasil e na maioria dos países europeus são necessários exames complementares, estes geralmente não são exigidos nos protocolos norte-americanos.^(4)^

No Brasil, de janeiro a dezembro de 2017, foram notificados 10.629 potenciais doadores (pacientes diagnosticados com ME e sem contraindicações à doação de órgãos). No entanto, apenas 3.415 indivíduos (32%) tornaram-se doadores efetivos.^(5)^ Os principais obstáculos a um maior número de doações de órgãos no Brasil podem ser atribuídos à falha na identificação de possíveis doadores, à recusa da família e a contraindicações clínicas.^(6)^ Em 2017, houve uma atualização da lei brasileira sobre a declaração de ME (decreto 9.175, de 18 de outubro de 2017, e Resolução do Conselho Federal de Medicina 2.173/17, de 23 de novembro de 2017), com o objetivo de aumentar o número de doações bem-sucedidas de órgãos^(7,8)^ – Westphal et al., oferecem abrangente análise abrangente dessas mudanças.^(9)^

Uma doação bem-sucedida de órgãos está associada a um breve intervalo de tempo entre o primeiro e o segundo exame clínico para ME.^(10)^ A redução dos intervalos de tempo na declaração de ME provavelmente seria uma boa estratégia para aumentar o número de órgãos e tecidos disponíveis para transplante.

## OBJETIVO

Caracterizar os processos de diagnóstico de morte encefálica e doação de órgãos em um centro de referência em trauma; verificar o perfil dos pacientes que tiveram o diagnóstico de morte encefálica; avaliar o impacto dos intervalos de tempo da declaração de morte encefálica na doação de órgãos; e checar os preditores de uma doação bem-sucedida de órgãos.

## MÉTODOS

### Delineamento e sujeitos do estudo

Estudo transversal realizado no Hospital Metropolitano de Urgência e Emergência, um centro de referência em trauma de Belém, (PA), Brasil. Os dados foram obtidos por meio da compilação de prontuários e formulários de declaração de ME em documentos da Organização de Procura de Órgãos (OPO) de pacientes diagnosticados com ME entre junho de 2014 e junho de 2017. Incluímos todos os pacientes com idade acima de 2 anos e excluímos os pacientes sem dados referentes a informações essenciais da internação hospitalar. O estudo foi aprovado pelo Comitê de Ética em Pesquisa da Universidade do Estado do Pará (CAAE: 65663717.0.0000.5174, parecer: 1.992.783), e como todos os pacientes eram falecidos, o Termo de Consentimento Livre e Esclarecido foi dispensado.

Como a coleta de dados ocorreu antes da nova lei brasileira sobre a declaração de ME, todos os casos seguiram a legislação anterior (lei 9.434, de 4 de fevereiro de 1997, e Resolução do Conselho Federal de Medicina 1.480/97).^(11,12)^

### Avaliações

Um protocolo de pesquisa específico foi utilizado para registrar dados sobre as variáveis clínicas e epidemiológicas (idade, sexo, escore da escala de coma de Glasgow na admissão e causa da morte) e sobre as etapas da declaração de ME. Definiu-se ME iminente na admissão hospitalar de acordo com de Groot et al.^(13)^ Utilizamos as seguintes variáveis de tempo como medidas indiretas da efetividade da declaração de ME: tempo entre a internação hospitalar e a admissão na unidade de terapia intensiva (UTI), tempo entre a admissão na UTI e o primeiro exame clínico de ME, tempo entre o primeiro e o segundo exame clínico de ME, e tempo entre o segundo exame clínico de ME e o exame complementar.

Dividimos os desfechos de doação de órgãos em estritos (doações “bem-sucedidas” ou “malsucedidas”) e amplos (“bem-sucedidas”, “malsucedidas por recusa familiar”, “malsucedidas por parada cardíaca” e “malsucedidas por outras causas”). “Outras causas” incluiu pacientes excluídos por normas médicas (não adequados para doação de órgãos) e outros motivos. Definimos “doação de órgão bem-sucedida” quando um órgão foi coletado de um paciente com ME para transplante.

### Análise estatística

O software Microsoft Excel 2010 foi usado para compilar as folhas de dados. O teste de Shapiro-Wilk foi utilizado para avaliar a normalidade das amostras. Para avaliar as variáveis categóricas, foram utilizados o teste χ^2^ e o teste exato de Fisher. Para variáveis contínuas, foi utilizado o teste de Mann-Whitney para duas amostras independentes e o teste de Kruskal-Wallis para três ou mais amostras independentes. Foram realizadas análises de regressão logística univariada para os possíveis preditores de uma doação de órgãos bem-sucedida, com o desfecho estrito de doação de órgãos (“bem-sucedida” ou “malsucedida”) como variável dependente. Todas as inferências estatísticas foram calculadas no (SPSS) for Windows, versão 23.0 (SPSS Inc., Chicago, USA), adotando como critério de significância valor de p≤0,05.

## RESULTADOS

### Caraterísticas clínicas e epidemiológicas

Durante 3 anos, foram notificados 159 pacientes com ME (Tabela 1). A maioria dos pacientes com ME era do sexo masculino (82,6%), adultos jovens (idade entre 18 e 40 anos; n=97; 61%) e vítimas de lesão encefálica traumática (93,7%), conforme esperado em um centro de referência em trauma. Crianças e adolescentes representaram 17,6% (n=28). Aproximadamente um terço dos pacientes apresentava ME iminente na admissão hospitalar (34,8%), sem impacto na doação de órgãos (χ^2^=0,22; p=0,71). O segundo exame clínico foi realizado geralmente por neurologistas/neurocirurgiões (72,3%), e o número de doações de órgãos bem-sucedidas foi maior (41,1%) quando o primeiro exame clínico foi feito por neurologista/neurocirurgião (χ^2^=4,18; p=0,04). Todos os casos determinados como ME no primeiro exame clínico foram confirmados pelo segundo exame clínico. O Doppler transcraniano foi o exame complementar escolhido em todos os pacientes.

**Tabela 1:** Característica gerais dos pacientes com morte encefálica por desfechos estritos de doação de órgãos

Características gerais	Geral (n=159)	Doação bem-sucedida (n=56)	Doação malsucedida (n=103)	Valor de p
Sexo masculino	137 (86,2)	49 (87,5)	88 (85,4)	0,459^*^
Idade, anos^†^	28 (20-53)	27 (21-42)	28 (20-40)	0,054^‡^
ME iminente na admissão hospitalar	49 (34,8)	19 (37,3)	30 (33,3)	0,386^*^
Causa de morte				0,055^*^
Lesão encefálica traumática	149 (93,7)	56 (100,0)	93 (90,3)	
Acidente vascular cerebral	9 (5,7)	0	9 (8,7)	
Causas metabólicas	1 (0,6)	0	1 (1,0)	
Neurologista/neurocirurgião como examinador				
No primeiro exame clínico de ME	48 (31,0)	23 (41,1)	25 (25,3)	0,041^*^
No segundo exame clínico de ME	107 (72,3)	35 (64,8)	72 (76,6)	0,123^*^
Tempo entre a admissão hospitalar e a admissão na UTI, horas^†^	19,8 (11-47)	20,5 (11-48)	19 (10-48)	0,855^‡^
Tempo entre a admissão na UTI e o primeiro exame clínico de ME, horas^†^	86,6 (43-159)	85 (47-190)	88,2 (41-132)	0,967^‡^
Tempo entre o primeiro e o segundo exame clínico de ME, horas^†^	10,5 (7-24)	12,4 (7-24)	9 (7-16)	0,878^‡^
Tempo entre o segundo exame clínico de ME e o exame complementar, horas^†^	10,7 (3-17)	9,8 (2-19)	10,7 (4-15)	0,176^‡^

Resultados expressos como n (%) ou mediana (intervalo interquartil).

* Teste χ^2^ comparando a frequência de doações de órgãos bem-sucedidas e malsucedidas; ^†^ Valores em mediana (intervalo interquartil); ^‡^ Teste de Mann-Whitney comparando as medianas de doações de órgãos bem-sucedidas e malsucedidas.

ME: morte encefálica; UTI: unidade de terapia intensiva.

### Impacto dos intervalos de tempo para a declaração de morte encefálica na doação de órgãos

O intervalo mediano total da declaração de ME (do primeiro exame clínico de ME ao exame complementar final) foi de 20,75 horas (intervalo interquartil 14-29), sem diferença entre os pacientes com ME e doações de órgãos bem-sucedidas e malsucedidas (teste de Mann-Whitney =2.075; p=0,479) Não houve diferença entre os quatro intervalos de tempo analisados (tempo entre a admissão hospitalar e a admissão na UTI, tempo entre a admissão na UTI e o primeiro exame clínico de ME, tempo entre o primeiro e o segundo exame clínico de ME, e tempo entre o segundo exame clínico de ME e o exame complementar) entre os pacientes com ME com doações de órgãos bem-sucedidas e malsucedidas. Tivemos longos intervalos de tempo antes do primeiro exame clínico de ME, principalmente no tocante ao tempo entre a admissão na UTI e o primeiro exame clínico de ME (mediana de 86,6 horas). Somente metade dos pacientes teve um tempo entre o primeiro e o segundo exame clínico de ME de aproximadamente 6 a 12 horas, conforme recomendado pela resolução anterior do Conselho Federal de Medicina^(11,12)^ (Figura 1).

**Figura 1:** Fluxograma de distribuição de pacientes com morte encefálica de acordo com o tempo entre o primeiro e o segundo exames clínicos

Analisamos se os diferentes intervalos de tempo poderiam aumentar ou reduzir o número de doações de órgãos bem-sucedidas. Para isso, usamos duas variáveis: o tempo entre o primeiro e o segundo exame clínico de ME, e o tempo entre o segundo exame clínico de ME e o exame complementar. As duas variáveis de intervalo de tempo foram agrupadas em quatro quartis, e as proporções de desfechos amplos de doação de órgãos foram distribuídas nos grupos de intervalos de tempo (Tabela 2). Em relação ao tempo entre o primeiro e o segundo exame clínico de ME, não houve diferença nas doações de órgãos bem-sucedidas entre o intervalo mais curto (<7 horas) e o intervalo mais longo (>15,5 horas; χ^2^=9,86; p=0,36), e a proporção de doações de órgãos malsucedidas por recusa familiar também foi semelhante entre os intervalos de tempo extremos. No tocante ao tempo entre o segundo exame clínico de ME e o exame complementar, a proporção de doações de órgãos bem-sucedidas diminuiu entre o intervalos mais curto (<2,5 horas) e o intervalo mais longo (>15 horas), e a proporção de doações de órgãos malsucedidas por recusa familiar aumentou inversamente; no entanto, essas diferenças não foram estatisticamente significativas (χ^2^=6,25; p=0,71).

**Tabela 2:** Efeito dos intervalos de tempo para a declaração de morte encefálica nos desfechos amplos de doação de órgãos

	Tempo entre o primeiro e o segundo exame clínico de ME				
Desfechos de doação de órgãos	<7 horas	7-9,5 horas	9,5-15,5 horas	>15,5 horas	Total
Bem-sucedida	16 (44,4)	9 (23,1)	13 (34,2)	15 (40,5)	53 (35,3)
Malsucedida – recusa da família	17 (47,2)	25 (64,1)	18 (47,4)	15 (40,5)	75 (50,0)
Bem-sucedida – parada cardíaca	0	2 (5,1)	3 (7,9)	1 (2,7)	6 (4,0)
Malsucedida – outras causas	3 (8,3)	3 (7,7)	4 (10,5)	6 (16,2)	16 (10,7)
Total	36 (24,0)	39 (26,0)	38 (25,3)	37 (24,7)	150 (100,0)
	**Tempo entre o segundo exame clínico de ME e o exame complementar**				
Desfechos de doação de órgãos	<2,5 horas	2,5-7 horas	7-15 horas	>15 horas	Total
Bem-sucedida	13 (40,6)	18 (46,2)	12 (36,4)	11 (30,6)	54 (38,6)
Malsucedida – recusa da família	15 (46,9)	16 (41,0)	17 (51,5)	21 (58,3)	69 (49,3)
Bem-sucedida – parada cardíaca	3 (9,4)	1 (2,6)	1 (3,0)	1 (2,8)	6 (4,3)
Malsucedida – outras causas	1 (3,1)	4 (10,3)	3 (9,1)	3 (8,3)	11 (7,9)
Total	32 (22,9)	39 (27,9)	33 (23,6)	36 (25,7)	140 (100,0)

Resultados expressos por n (%).

Os valores representam a proporção e o número de pacientes com morte encefálica distribuídos entre os quartis de intervalos de tempo (tempo entre os dois exames clínicos, e tempo entre o segundo exame clínico e o exame complementar), de acordo com os desfechos amplos de doação de órgãos.

ME: morte encefálica.

### Preditores de doação de órgãos bem-sucedida após morte encefálica

Foram realizadas análises de regressão logística univariada para o papel dos preditores (idade, sexo, escore na escala de coma Glasgow na admissão e intervalos de tempo) nos desfechos estritos de doação de órgãos, e apenas a idade foi inversamente associada à probabilidade de uma doação de órgãos bem-sucedida (coeficiente B -0,027; razão de chances 0,973; intervalo de confiança de 95% 0,94-0,99; p=0,027) (Tabela 3).

**Tabela 3:** Análise univariada de preditores de doação de órgãos bem-sucedida após morte encefálica

Preditores	B	RC	IC95%	Valor de p
Sexo				
Feminino	Referência			
Masculino	0,177	1,19	0,45-3,12	0,719
Idade no momento do estudo	-0,027	0,97	0,94-0,99	0,027
Escala de coma de Glasgow 3 na admissão	0,172	1,18	0,58-2,43	0,639
Tempo entre a admissão hospitalar e a admissão na UTI	0,176	1,19	0,4-3,48	0,747
Tempo entre a admissão na UTI e o primeiro exame clínico de ME	0,184	1,2	0,36-3,95	0,763
Tempo entre o primeiro e o segundo exame clínico de ME	0,051	1,05	0,29-3,7	0,937
Tempo entre o segundo exame clínico de ME e o exame complementar	-0,234	0,79	0,43-1,44	0,444

B: coeficiente de regressão; ME: morte encefálica; RC: razão de chances; IC95%: intervalo de confiança de 95%.

## DISCUSSÃO

Os presentes resultados mostraram que nossos pacientes com ME eram, em sua maioria, jovens adultos do sexo masculino que sofreram lesões encefálicas traumáticas. Estudos realizados no Brasil descreveram jovens do sexo masculino como o principal grupo associado a ME, mas a lesão encefálica traumática não foi a principal causa de morte desses pacientes.^(14-17)^ Como nossa amostra foi coletada em um centro de referência em trauma, nossos resultados podem ter um viés de seleção.

Cerca de um terço desses pacientes havia sido internado em ME iminente, um conceito criado para aprimorar o reconhecimento clínico de potenciais doadores de órgãos, com alta sensibilidade e baixa especificidade para prever ME.^(13,18)^ Em nossa amostra, a internação hospitalar com escore 3 na escala de coma de Glasgow não influenciou no número de doações de órgãos bem-sucedidas. Se medidas específicas de elucidação da ME para a família e terapia intensiva fossem usadas em pacientes com ME imediata, o número de doadores poderia ser maior.

Os intervalos de tempo para a declaração de ME em nosso estudo foram maiores do que o esperado, e atribuímos esse fato a problemas específicos do centro de referência (poucos neurologistas e neurocirurgiões, baixo nível de treinamento médico na declaração de ME, e baixa disponibilidade de exames complementares). Esses dados ajudaram a organização a melhorar seu protocolo de declaração de ME e a disponibilizar exames complementares.

No entanto, existe uma grande variabilidade em relação a esses intervalos de tempo em todo o mundo. Estudos anteriores descreveram um tempo entre o primeiro e o segundo exame clínico de ME de 8,9 horas^(17)^ a 19,2 horas,^(10)^ e o intervalo de tempo total da declaração de ME foi relatado como sendo de 14,4 horas.^(17)^ Um maior intervalo de tempo total da declaração de ME está associado a mais paradas cardíacas,^(19)^ maiores custos da terapia intensiva e pior qualidade dos órgãos destinados a transplante,^(20,21)^ e um menor intervalo entre a admissão e a declaração de ME aumenta o número de órgãos transplantados por doador.^(22)^ Além disso, um tempo de isquemia fria maior do que 24 horas está associado à menor sobrevida do enxerto renal.^(23)^

Devemos levar em consideração a grande variabilidade no processo de declaração de ME em todo o mundo.^(4)^ Revisão recente relatou baixa homogeneidade no processo de declaração de ME em 91 países, com diferentes protocolos para teste de apneia, necessidade e tipo de exame complementar, número e qualificação de médicos, e critérios utilizados em crianças.^(24)^

Nosso estudo mostrou que o tempo entre o primeiro e o segundo exame clínico de ME não teve efeito no desfecho de doação de órgãos, tanto na comparação da proporção de doadores em quartis de intervalo distintos quanto na regressão logística univariada. Esse resultado difere das conclusões de estudo anterior, que analisou o efeito desse intervalo de tempo na doação de órgãos em 1.311 pacientes com ME.^(10)^ Eles demonstraram que intervalos mais longos entre os dois exames de ME causaram redução na proporção de doadores, aumento na recusa familiar de doação de órgãos, e mais paradas cardíacas. Atribuímos esses resultados divergentes a diferenças no tamanho da amostra e na variabilidade da metodologia de declaração da ME.

Nosso estudo tem limitações importantes. O pequeno tamanho de amostra e o recrutamento de um único centro que recebe principalmente pacientes traumatizados podem prejudicar a capacidade de generalização dos resultados. A baixa proporção de doadores (35,2%) também pode interferir nos resultados finais. Como ponto forte, nosso trabalho é o primeiro estudo brasileiro que explorou os intervalos de tempo para a declaração do ME e seu impacto na doação de órgãos.

## CONCLUSÃO

Foi baixa a eficácia da declaração de morte encefálica com base em intervalos de tempo mais longos para essa declaração, sem impacto na doação de órgãos. A redução do tempo entre os dois exames clínicos de morte encefálica e a rápida execução de exames complementares devem ser prioritárias nos hospitais para melhorar a qualidade da declaração de morte encefálica.
